# Development of early diagnosis of Parkinson's disease: Illusion or reality?

**DOI:** 10.1111/cns.13429

**Published:** 2020-06-29

**Authors:** Michael Ugrumov

**Affiliations:** ^1^ Laboratory of Neural and Neuroendocrine Regulations Institute of Developmental Biology RAS Moscow Russia

**Keywords:** animal models, biomarkers, early diagnosis, neurodegeneration, Parkinson's disease

## Abstract

The fight against neurodegenerative diseases, Alzheimer disease and Parkinson's disease (PD), is a challenge of the 21st century. The low efficacy of treating patients is due to the late diagnosis and start of therapy, after the degeneration of most specific neurons and depletion of neuroplasticity. It is believed that the development of early diagnosis (ED) and preventive treatment will delay the onset of specific symptoms. This review evaluates methodologies for developing ED of PD. Since PD is a systemic disease, and the degeneration of certain neurons precedes that of nigrostriatal dopaminergic neurons that control motor function, the current methodology is based on searching biomarkers, such as premotor symptoms and changes in body fluids (BF) in patients. However, all attempts to develop ED were unsuccessful. Therefore, it is proposed to enhance the current methodology by (i) selecting among biomarkers found in BF in patients at the clinical stage those that are characteristics of animal models of the preclinical stage, (ii) searching biomarkers in BF in subjects at the prodromal stage, selected by detecting premotor symptoms and failure of the nigrostriatal dopaminergic system. Moreover, a new methodology was proposed for the development of ED of PD using a provocative test, which is successfully used in internal medicine.

## Graphical abstract

Timing characteristics of Parkinson's disease with a focus on the degradation of nigrostriatal dopaminergic system, responsible for the regulation of motor function. Main methodologies for developing early diagnosis of Parkinson's disease: (i) searching for premotor symptoms at the prodromal stage; (ii) searching for biomarkers in body fluids and peripheral tissues at the preclinical and prodromal stages; (iii) provocative test at the preclinical and prodromal stages.

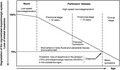

## 1 INTRODUCTION

One of the global challenges in the XXI century is the fight against socially significant neurodegenerative diseases, mainly Alzheimer disease and Parkinson's disease (PD), which is due to a rapidly growing incidence and high cost of treatment and rehabilitation.^1^, ^2^, ^3^. Neurodegenerative diseases are diagnosed a long time after the onset, under degeneration of most specific neurons and the depletion of brain neuroplasticity, by the appearance of specific symptoms.^4^, ^5^, ^6^. This explains the low efficacy of current symptomatic therapy.^2^, ^7^, ^8^, ^9^, ^10^, ^11^ It is believed that the development of early (preclinical) diagnosis (ED) and preventive therapy can prolong the preclinical stage of a patient's comfortable life.^12^ However, despite great efforts, ED of neurodegenerative diseases has not yet been developed. This casts doubt on the adequacy of the methodology used for its development.^13^ Therefore, this review aims to evaluate the experience of developing ED of PD using the current methodology, as well as the prospect of enhancing its efficacy or using a new methodology.

## 2 ETIOLOGY, PATHOGENESIS, AND MANIFESTATION OF PARKINSON'S DISEASE AS A BASIS FOR THE DEVELOPMENT OF EARLY DIAGNOSIS

### 2.1 Etiology

Less than 10% of patients suffer from monogenic PD, which is predetermined by modifications of risk genes, Parkin, DJ‐1, PINK1 ATP13A2, DNAJC6, PLA2G6, FBOX7 and SYNJ1, SNCA, LRRK2, VPS35, etc.^14^, ^15^, ^16^, ^17^, ^18^, ^19^, ^20^ However, most patients suffer from idiopathic, or sporadic PD, a multifactorial disease caused by a combination of genetic and epigenetic factors (age, environment, lifestyle, brain injury, etc.).^8^, ^17^, ^21^, ^22^, ^23^, ^24^ This review focuses on sporadic PD. The development of sporadic PD is promoted by toxic factors that affect central and peripheral neurons, including nigrostriatal dopaminergic neurons, which control motor function.

Endogenous toxins are mainly represented by neuron‐derived misfolded aggregated proteins, synuclein and tau,^19^, ^25^, ^26^ as well as proinflammatory cytokines secreted by T lymphocytes and glia.^27^, ^28^ Along with neurotoxins, neuroprotectors are produced in PD. The most effective are antioxidants, such as DJ‐1 protein, which is encoded by the PARK7 gene, and urates secreted by the liver and intestines.^19^, ^29^, ^30^

### 2.2 Pathogenesis

**FIGURE 1:**
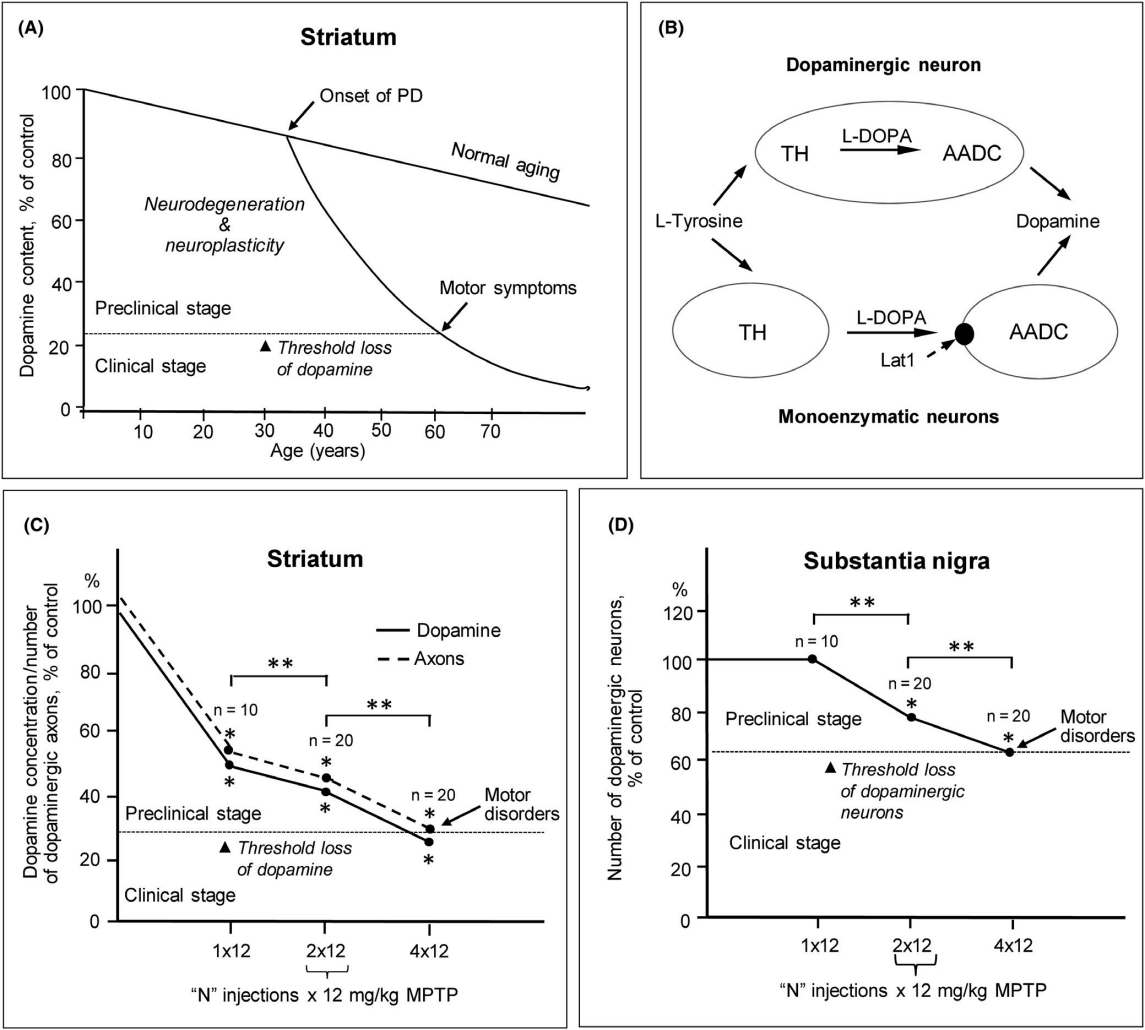
Schematic representation of the pathogenesis of Parkinson's disease (PD) in patients and its reproducing in animal models. A, The manifestation of progressive degradation of the nigrostriatal dopaminergic system in patients with PD as a loss of dopamine (DA) in the striatum to a threshold level, which is associated with the onset of motor disorders (modified from^31^); B, The main pathways of the neuroplasticity in PD, represented by a compensatory increase in DA synthesis in survived nigrostriatal dopaminergic neurons and the onset of DA synthesis in non‐dopaminergic neurons, expressing only tyrosine hydroxylase (TH) or only aromatic L‐amino acid decarboxylase (AADC) (modified from^52^); C, D, Progressive loss of DA and dopaminergic axons in the striatum (C), as well as dopaminergic neurons (cell bodies) (D) in the substantia nigra when modeling preclinical and clinical stages of PD in mice by subcutaneous injections once, twice or four times 1‐methyl‐4‐phenyl‐1,2,3,6‐tetrahydropyridine (MPTP) at the individual dose of 12 mg/kg with an interval of two hours between injections. Control animals received 0.9% NaCl instead of MPTP (modified from^118^). The data were treated with *F*‐test to compare variances and with Student's *t* test for the estimation of statistically significant differences. Both tests are included in Sigma Plot version 9.1 and GraphPad Prism version 5.0, software packages for Windows (GraphPad Software, USA). Data are represented as the mean ± SEM. *p < 0.05 statistically significant differences compared to the control; **p < 0.05, for differences between selected parameters. Lat1, membrane transporter of large neutral L‐amino acids and L‐DOPA; n, number of animals in the experiment and the same in the control

The pathogenesis of sporadic PD is characterized by a number of hallmarks. First, PD develops up to 30 years at the preclinical stage without manifestations of motor disorders. They appear after a “threshold” degradation of the nigrostriatal dopaminergic system at a loss of 70% dopamine in the striatum and 50‐60% dopaminergic neurons in the substantia nigra (Figure 1A).^5^, ^8^, ^22^, ^31^ Second, neurodegeneration, which is usually caused by oxidative stress due to mitochondria pathology^6^, ^15^, ^32^ is not limited to the nigrostriatal dopaminergic system. It extends to other central and peripheral neurons.^33^, ^34^, ^35^ Third, neurodegeneration is enhanced due to the impairment of the blood‐brain barrier.^36^, ^37^ Fourth, the neurodegeneration of some central and peripheral neurons precedes the neurodegeneration of dopaminergic neurons.^22^, ^33^

A key molecular mechanism of PD progression is associated with metabolic abnormalities of α‐synuclein, which is normally involved in the control of mitochondrial function and neurotransmission.^15^ It becomes toxic as a result of intraneuronal accumulation due to an impaired proteasomal degradation by ubiquitin‐C‐terminal hydrolase‐L1 and β‐glucocerebrosidase,^25^, ^38^, ^39^ followed by aggregation, misfolding, and phosphorylation at serine‐129.^40^ Although toxic α‐synuclein affects all neurons, it appears to provide a semi‐specific effect on nigrostriatal dopaminergic neurons, probably enhancing the cytosolic dopamine autotoxicity.^11^, ^41^ Tau proteinopathy, like α‐synucleinopathy, promotes neurodegeneration due to impaired axonal transport in affected neurons, including nigrostriatal dopaminergic neurons. This leads to impaired neurotransmission and degradation of synaptic terminals. In addition to toxic proteins, miRNAs are involved in the PD pathogenesis, enhancing neurodegeneration through regulation of risk genes.^16^, ^19^, ^42^, ^43^

In addition to endogenous neurotoxins, some environmental factors contribute to the PD pathogenesis. So, microbiota toxins upon entry into the body promote α‐synucleinopathy and the spread of an abnormal α‐synuclein through the body in a prion‐like manner.^24^, ^44^, ^45^ In turn, heavy metals that enter intranasally in the brain accumulate in dopaminergic neurons of the substantia nigra, causing oxidative stress and neurodegeneration.^16^, ^24^ The toxicity of some exogenous factors depends on the genetic polymorphism of cytochrome P450 2D6 (CYP2D6), an enzyme involved in their metabolism. In fact, the risk of developing PD under the influence of pesticides increases at CYP2D6 deficiency.^46^

Functional deficiency in PD patients is compensated by brain neuroplasticity, which explains long‐term development of the disease without manifestation of motor symptoms (Figure 1B).^4^, ^5^, ^47^, ^48^, ^49^, ^50^ Compensatory processes include the following: (i) an increase in the functional activity of survived dopaminergic neurons, (ii) dopamine synthesis by striatal non‐dopaminergic neurons expressing only tyrosine hydroxylase or aromatic L‐amino acid decarboxylase (Figure 1B), (iii) increased sensitivity of target neurons to dopamine.^4^, ^5^, ^47^, ^49^, ^50^, ^51^, ^52^, ^53^ In addition to the specific mechanisms of neuroplasticity that maintain dopamine neurotransmission at a normal level, there are nonspecific compensatory processes mediated by growth factors. These include the following: (i) axon branching and multiplication of synaptic terminals, (ii) activation of antioxidant systems, (iii) elimination of neurotoxins by glial cells.^54^, ^55^

### 2.3 Clinical manifestations, diagnosis

According to the last classification, three stages are distinguished in PD development: (i) the preclinical stage—from the onset of neurodegeneration to the appearance of nonmotor symptoms, (ii) the prodromal stage—from the onset of nonmotor symptoms to the appearance of motor symptoms, bradykinesia, rigidity, rest tremor, (iii) clinical stage—from the appearance of motor symptoms to death.^2^, ^9^, ^23^, ^24^, ^56^, ^57^, ^58^, ^59^

## 3 CURRENT METHODOLOGY FOR DEVELOPING EARLY DIAGNOSIS OF PARKINSON'S DISEASE

Low efficacy of the symptomatic treatment of PD due to its late start makes it necessary to develop ED and preventive treatment. The current methodology for the development of ED is based, on the one hand, on searching biomarkers as premotor symptoms and demonstration of the failure of the nigrostriatal dopaminergic system using positron emission tomography (PET) during so‐called prodromal phase, corresponding to a 10‐year period before the onset of motor disorders.^2^, ^13^, ^60^ On the other hand, this methodology is also based on the search for biomarkers as changes in body fluids (BF), which can be detected not only in the prodromal phase, but also in the preceding preclinical phase.^2^, ^13^ Such biomarkers are defined by the National Institutes of Health as a characteristic that is objectively measured and evaluated as an indicator of normal biologic processes, pathogenic processes, or pharmacologic responses to a therapeutic intervention.^61^ In this paper, when referring to the early or preclinical diagnosis, we mean a diagnosis at the prodromal and preclinical stages of PD by Postuma and Berg.^2^

### 3.1 Noninvasive neuroimaging

PD can already be diagnosed at the prodromal stage with PET, by detecting decreased synthesis, uptake, vesicular storing, and release of dopamine in the nigrostriatal dopaminergic system.^13^, ^17^, ^58^, ^62^, ^63^, ^64^ However, this approach cannot be used for preventive examination of a healthy population because of the high cost. Although spectroscopic magnetic resonance imaging and transcranial sonography can also be used for ED of PD, their predictive value is low (3%).^58^, ^64^, ^65^

### 3.2 Search for premotor symptoms

Early biomarkers of PD include premotor symptoms that result from the degeneration of certain central and peripheral neurons, prior to the degeneration of nigrostriatal dopaminergic neurons.^21^, ^22^, ^23^, ^33^, ^35^, ^60^, ^66^ Initially, premotor symptoms were identified by retrospective analysis of the medical history, but later this methodology has been enhanced by detecting premotor symptoms in elderly people without motor disorders.^19^, ^23^, ^60^, ^66^, ^67^, ^68^ The diagnostic value of premotor symptoms in subjects at risk is confirmed by the detection of failure of the nigrostriatal dopaminergic system using PET^2^, ^19^, ^58^ and then by the appearance of motor disorders.^2^, ^58^, ^60^, ^64^, ^68^ The most obvious premotor symptoms of PD are rapid eye movement (REM) sleep behavior disorder, characterized by vivid, intense, and violent sleep activity, as well as impaired olfaction. It should be noted that patients with PD do not realize that their sense of smell is impaired. Detection of olfactory impairment became possible due to the development of clinical olfactory tests (the University of Pennsylvania Smell Identification Test) in the 80s.^69^ It was shown that 67% of elderly people with impaired olfaction or rapid eye movement (REM) sleep behavior disorder and a failure of the nigrostriatal dopaminergic system manifest motor symptoms within 4 years.^70^, ^71^

In addition to premotor symptoms due to brain pathology, premotor symptoms such as constipation are the result of autonomic dysfunctions.^2^, ^66^ However, a prognostic value of constipation is low, since its prevalence among the whole population is only 15‐20%.^2^ Despite some progress in identifying premotor symptoms, their use for ED of PD in a preventive examination of a healthy population is problematic, since premotor symptoms are not specific and their detection (somnography, PET) is expensive.^72^

### 3.3 Targeted search for biomarkers in body fluids

Besides premotor symptoms, changes in BF are considered as potential biomarkers for ED of PD.^63^, ^73^ However, due to the inability to diagnose PD at the preclinical stage, biomarkers are usually searched in BF in untreated PD patients at the clinical stage. Although dozens of studies have attempted to identify biomarkers in BF, most of the results are contradictory. Nevertheless, some changes in the level of endogenous neurotoxins, classical neurotransmitters and their metabolites, lipids, products of oxidative stress, inflammatory cytokines, microRNAs, and specific antibodies in BF are considered as biomarkers of PD.^13^, ^14^, ^42^, ^74^ Most often, biomarkers are detected in the cerebrospinal fluid (CSF) and in the blood, but their content in these BF is different, which is probably due to the presence of the blood‐brain barrier, despite the fact that its permeability increases in neurodegenerative diseases.^36^, ^37^

Given the crucial role of α‐synuclein in PD pathogenesis, particular attention is paid to its detection in CSF and plasma. It was shown that the concentration of total α‐synuclein decreased in CSF and plasma in PD patients, whereas the concentrations of toxic phosphorylated at serine‐129 and oligomeric α‐synuclein increased in both BF compared to age‐matched control.^19^, ^40^, ^75^, ^76^, ^77^, ^78^ The sensitivity and specificity of the PD diagnosis increase up to 89.3% and 90.6%, when assessing the ratio of α‐synuclein oligomers to the total content of α‐synuclein.^13^, ^21^, ^75^ Aggregated and phosphorylated tau protein is also considered as a biomarker, since their concentrations decrease in CSF in PD patients.^78^, ^79^ The formation of toxic isoforms of synuclein and tau in PD is due to insufficiency of the proteasome system, which is manifested in a decrease in the level of ubiquitin‐C‐terminal hydroxylase‐L1 in CSF. The diagnostic sensitivity and specificity of this indicator is 89% and 67%, respectively.^19^ Moreover, the activity of ß‐glucocerebrosidase decreases in CSF in PD. The diagnostic accuracy of PD increases, when assessing a combination of biomarkers, for example, a ratio oligomeric‐α‐synuclein/total‐α‐synuclein and activity of ß‐glucocerebrosidase in CSF.^19^, ^75^, ^80^ Along with proteinopathies, neuroinflammation contributes to the progression of PD, which is associated with an increased level of toxic cytokines in CSF and plasma.^13^, ^19^, ^43^, ^81^, ^82^ MicroRNAs, that control the expression of risk genes and are contained in CSF in free form and in exosomes, are also considered PD biomarkers.^16^, ^83^ The diagnostic sensitivity of the miRNAs panel reaches 90%.^84^

Since neurodegeneration is associated with neuroplasticity, some biomarkers of PD are represented by endogenous neuroprotectors, mainly antioxidants. In fact, the level of some DJ‐1 isoforms, especially 4‐hydroxy‐2‐nonenal, is lower in CSF and plasma in PD patients compared to age‐matched controls.^19^, ^21^ Another antioxidant, urate, is also a biomarker of PD, as its plasma concentration in patients is lower than in elderly control, but higher than in CSF of patients.^19^, ^29^ Some growth factors are also considered as neuroprotective biomarkers of PD. The level of BDNF, transforming growth factor, insulin‐like growth factor, and epidermal growth factor increased in CSF, whereas the level of BDNF in plasma decreased in PD patients compared to healthy control.^82^, ^85^

Although the degeneration of neurons producing monoamines or amino acid neurotransmitters is characteristic of PD, data on changes in the content of these neurotransmitters and metabolites in CSF and plasma are contradictory.^82^, ^86^, ^87^, ^88^, ^89^, ^90^, ^91^, ^92^, ^93^, ^94^, ^95^ This may be due to the fact that the level of monoamines in CSF and plasma is determined by the neurodegeneration and neuroplasticity of the corresponding neurons, which should differ at different stages of pathogenesis.

In addition to CSF and plasma, PD biomarkers were found in saliva and urine.^40^, ^96^, ^97^, ^98^ It should be emphasized that, although the individual biomarkers found so far in PD patients are not specific, the accuracy of ED can be increased by using a combination of various diagnostic biomarkers.^13^, ^21^, ^72^, ^80^

In addition to plasma biomarkers, blood biomarkers of PD include changes in the expression of specific genes and phenotype of blood cells due to changes in the environment (plasma). Indeed, lymphocytes in PD are characterized by a modification of gene expression of receptors for dopamine.^95^, ^99^, ^100^ Moreover, lymphocytes in PD patients manifest a decreased content of the dopamine membrane transporter.^101^ Changes in gene expression and phenotype have also been detected in red blood cells. They show an increased ratio of oligomeric α‐synuclein to total α‐synuclein and an increased content of oxidized protein DJ‐1 in PD patients compared to age‐matched controls.^13^, ^17^, ^102^, ^103^

Searching for blood biomarkers has several advantages compared to searching in CSF: (i) the spectrum of plasma biomarkers is wider than in CSF, since they derive not only from the brain, but also from the periphery, (ii) blood sampling is easier and more safe than CSF sampling, (iii) blood biomarkers are represented not only by changes in plasma, but also in blood cells. Nevertheless, the specificity of biomarkers in CSF appears to be higher than those in the blood.

### 3.4 Targeted search for biomarkers in peripheral tissues

Lewy bodies (plaques of aggregated α‐synuclein), which are easily detected by biopsy in the gastrointestinal tract and salivary glands, are generally considered a reliable biomarker of PD.^11^, ^17^ However, the prospect of using this approach for ED is doubtful, since it is invasive, and Lewy bodies are not a specific attribute of PD.^104^, ^105^, ^106^, ^107^, ^108^ Another widely recognized peripheral biomarker of PD is fecal microbiome of the gastrointestinal tract.^17^, ^45^, ^63^

### 3.5 Nontargeted search for biomarkers of Parkinson's disease

Along with the use of targeted approaches to searching PD biomarkers in BF and peripheral tissues, non‐targeted OMIX methods were also used.^14^, ^109^ Although OMIX techniques gives an opportunity to detect changes in the content of thousands of proteins, low molecular weight compounds, lipids, as well as gene expression, no fundamentally new and specific diagnostic markers were found.^14^, ^21^, ^25^, ^42^, ^110^, ^111^

## 4 ENHANCED METHODOLOGY FOR DEVELOPING EARLY DIAGNOSIS OF PARKINSON'S DISEASE

As mentioned above, the most widely used methodology for developing ED of PD is based on searching for biomarkers such as premotor symptoms, changes in BF and deposition of Lewy bodies in peripheral tissues, which were found in untreated PD patients at the clinical stage. It is believed that biomarkers found in untreated PD patients at the clinical stage are characteristic of the preclinical stage, which is not the case. Indeed, given that pathological processes in PD gradually spread throughout the body, biomarkers at the preclinical stage should be at least less numerous than at the clinical stage. Therefore, it is tempting to develop an approach that makes it possible to recognize preclinical biomarkers among all biomarkers found at the clinical stage.

### 4.1 Searching for biomarkers in body fluids at the prodromal stage of Parkinson's disease

Significant progress in the development of ED of PD achieved by searching for premotor symptoms in elderly people without motor disorders, followed by confirmation of the failure of the nigrostriatal dopaminergic system, opens up the prospect of searching for preclinical biomarkers in BF at the prodromal stage. The use of such biomarkers in BF in combination with premotor symptoms could significantly increase the efficacy of ED and the accuracy of predicting the time of appearance of motor symptoms.^64^ However, there are only a few studies with using this approach.^22^, ^72^ Thus, it was shown that in subjects at risk with the REM sleep behavior disorder, there was a change in the blood level of complexin 1, a key protein of the vesicular cycle.^112^ Moreover, the subjects at risk, recognized by REM sleep behavior disorder or a failure of the nigrostriatal dopaminergic system with PET, have a reduced level of glutathione and apolipoprotein A‐1 in the blood.^113^, ^114^ Besides biomarkers in BF, Lewy bodies were found in the skin in subjects at risk, selected by the REM sleep behavior disorder.^115^ Although the use of biomarkers found in BF and peripheral tissues in subjects at risk may increase the selectivity and sensitivity of ED, it is likely to be non‐specific, since each of the known biomarkers is not specific.

### 4.2 Searching for biomarkers in body fluids in patients and animal models

The second approach to improve the methodology for the development of ED of PD can be based on the selection of hypothetical preclinical biomarkers among all biomarkers found in the blood of untreated PD patients at an early clinical stage. Apparently, this can be done using animal models of PD.^116^ According to our hypothesis, only those biomarkers can be considered as preclinical, which were found both in PD patients at the clinical stage and in animal models of the preclinical stage.^95^ However, first it is necessary to make sure that the selected model adequately reproduces the PD pathogenesis in certain metabolic pathways, showing the same marker in the blood of patients and animals when modeling the clinical stage of PD. To test this methodology, we used neurotoxic animal models of clinical and preclinical stages of PD, which are characterized by threshold and subthreshold degradation of the nigrostriatal dopaminergic system, respectively.^51^, ^52^, ^53^, ^117^, ^118^, ^119^, ^120^, ^121^ In this case, PD was reproduced in mice C57BL by systemic administration of 1‐methyl‐4‐phenyl‐1,2,3,6‐tetrahydropyridine (MPTP), converted in the body to 1‐methyl‐4‐phenylpyridinium, a toxin of dopaminergic neurons (Figure 1C,D).^116^, ^122^

Testing of this methodology showed that among 13 biomarkers found in untreated PD patients at the clinical stage, such as changes in plasma concentrations of catecholamines and amino acid neurotransmitters, as well as expression of specific genes in lymphocytes, 7 and 3 biomarkers were characteristics of models of PD at the clinical and preclinical stage, respectively.^95^ The hypothetical preclinical biomarkers of PD were represented by a reduced concentration of L‐DOPA and DOPAC in plasma and a change in the gene expression of a dopamine receptor (D3) in lymphocytes.^95^ This comparative analysis of the biomarkers in BF in PD patients and in animal models confirmed our hypothesis that only a small fraction of the biomarkers found in untreated PD patients at the early clinical stage can be used for ED. The final proof of the valuability of the proposed methodology for the development of ED of PD will be the appearance in subjects at risk selected by premotor symptoms, PET and biomarkers in BF, parkinsonian motor disorders over time.

Animal models of preclinical PD can be used not only to select preclinical biomarkers among all biomarkers that have already been found in PD patients, but also to searching biomarkers that were not been found in patients. Despite the fact that this idea was expressed several years ago,^21^ there is no progress.

Thus, the attempts to enhance the current methodology for developing ED of PD based on the search for biomarkers in BF appear to be promising. However, even with the most encouraging results, most biomarkers, if not all probably will not be specific, as ED based on their use.

## 5 NEW METHODOLOGY FOR DEVELOPING EARLY DIAGNOSIS OF PARKINSON'S DISEASE

The doubtful prospect of developing ED of PD based on searching for biomarkers, such as premotor symptoms and changes in BF, encourages the development of a fundamentally different, more specific methodology.

### 5.1 Provocative test as an approach to the early diagnosis of chronic diseases

The development of ED is a key point in the fight against chronic diseases not only in neurology and psychiatry, but also in internal medicine. This problem was successfully solved in internal medicine using a provocative test, or challenge test that is any procedure in which a suspected pathophysiological abnormality is deliberately enhanced by manipulations that cause a characteristic reaction or symptoms. This approach is also applicable to differential diagnosis.^123^, ^124^, ^125^, ^126^, ^127^, ^128^ Surprisingly, so far, there have been no attempts to use a provocative test for ED of chronic brain diseases.

Although various stimuli are used as a provocative test in internal medicine, pharmacological tests predominate.^123^, ^124^, ^127^, ^129^, ^130^ So, provocation using an intravenous or oral glucose tolerance test is widely applied to detect the abnormal regulation of glucose metabolism by insulin in diabetics.^131^, ^132^ A provocative test is also used to diagnose growth hormone deficiency, usually by assessing insulin tolerance or glucagon stimulation.^133^, ^134^ The list of chronic diseases of the internal organs diagnosed with a provocative test at the preclinical stage can be significantly expanded.

The use of a provocative test to detect latent failure of internal organs affected in PD is of particular interest. So, Nakamura et al^123^ developed a provocative test that determines cardiac desympathization, an attribute of PD. Indeed, dobutamine, a β1 receptor agonist, as a provocative factor, increases cardiac contractility, systolic blood pressure, and heart rate in PD to a greater extent than in norm, due to increased expression of adrenergic receptors under noradrenaline deficiency.^123^, ^135^

### 5.2 The use of a provocative test for early diagnosis of Parkinson's disease

**FIGURE 2:**
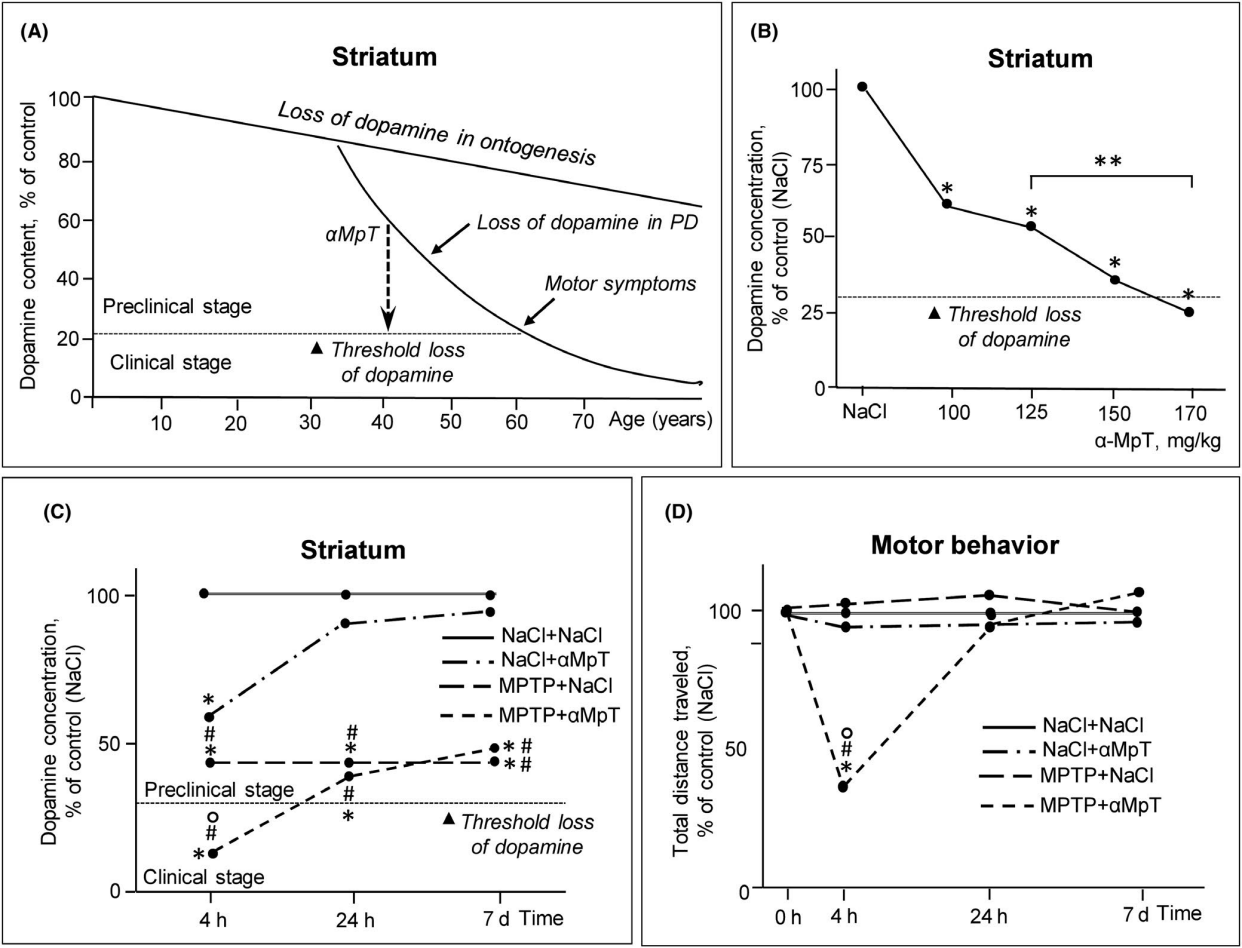
Implementation of a provocative test for the early (preclinical) diagnosis of Parkinson's disease (PD), using α‐methyl‐p‐tyrosine (αMpT), a nonmetabolizable reversible inhibitor of tyrosine hydroxylase and, hence, dopamine (DA) synthesis (modified from^134^). A, Schematic hypothetical representation of the depletion of striatal DA using αMpT in patients at the preclinical stage of PD to a threshold level at which motor disorders first appear; B, αMpT‐induced dose‐dependent depletion of DA in the striatum in normal mice 4 h after a single injection of αMpT. Values in control mice (0.9% NaCl) were taken as 100%. *p < 0.05 compared to the control, **p < 0.05, differences between selected parameters. For each animal group, n=9. Data are expressed as the mean ± SEM and were analyzed by the Kruskal‐Wallis test followed by Dunn's multiple comparisons test. C, DA concentration in the striatum of mice, received 0.9% NaCl (control) or MPTP (preclinical PD model) and two weeks later αMpT at the dose of 125 mg/kg or 0.9% NaCl (control). DA concentration in control mice (NaCl+NaCl group) was taken as 100%. *p < 0.05 compared to the control (NaCl+NaCl group); #p < 0.05 compared to NaCl+αMpT group; ^o^p < 0.05 compared to MPTP+NaCl group. For each group of mice, n=9. Data are expressed as the mean ± SEM and were analyzed by the two‐way ANOVA test by ranks followed by Tukey's multiple comparisons test. D, Basal locomotor activity of mice, received 0.9% NaCl (control) or MPTP (preclinical PD model) and two weeks later αMpT at the dose of 125 mg/kg or 0.9% NaCl. Values in control mice (NaCl+NaCl group) were taken as 100%. *p < 0.05 compared to the control (NaCl+NaCl group); #p < 0.05 compared to NaCl+αMpT group; ^o^p < 0.05 compared to MPTP+NaCl group. For each group of mice, n = 9. Data are expressed as the mean ± SEM and were analyzed by the two‐way ANOVA test by ranks followed by Tukey's multiple comparisons test

We believe that ED using a provocative test can be developed for any chronic brain disease associated with the degeneration of specific neurons and the loss of the corresponding neurotransmitter. The most suitable neurological disease for this development is apparently PD, since it was determined that motor symptoms appear at the threshold of degradation of the nigrostriatal dopaminergic system, with a loss of 70% dopamine in the striatum and 50‐60% dopaminergic neurons in the substantia nigra (Figure 1A).^31^, ^136^ We used α‐methyl‐p‐tyrosine (αMpT) as a provocative agent, which has a dose‐dependent reversible short‐term inhibitory effect on tyrosine hydroxylase and, hence, on dopamine synthesis (Figure 2A).^137^, ^138^ An initial study showed that αMpT at the dose of 170 mg/kg decreased the dopamine level in the striatum in intact mice to a threshold (73%), followed by impaired motor behavior, while at the dose of 125 mg/kg dopamine level reduced by 45%, and this did not lead to motor disorders (Figure 2B).^136^ Then, it was shown that the administration of αMpT at the dose of 125 mg/kg to mice at modeling the preclinical stage of PD caused a decrease in striatal dopamine to a superthreshold level, which caused motor disorders (Figure 2C,D). Twenty‐four hours or, probably, even earlier after the αMpT administration, the dopamine content in the striatum returned to a normal level, followed by normalization of motor behavior (Figure 2C,D). Moreover, a week after the application of the provocative test to animals at modeling preclinical stage of PD, there were no structural or functional changes in the nigrostriatal dopaminergic system compared with intact animals.^136^ These data show that αMpT does not induce long‐term side effects, as it is nonmetabolizable and fully eliminated from the body. Nevertheless, it should be taken in mind that αMpT, at systemic administration, decreases the content of catecholamines not only in the brain, but also in the peripheral nervous system, imitating the desympathization of internal organs, characteristic of PD. This complication can be avoided by intranasal administration of αMpT. In this case, αMpT is delivered directly to the brain, almost bypassing the general circulation.

Thus, it was shown for the first time that a provocative test can be potentially used for ED of PD.

### 5.3 Prospects and risks of using αMpT as a provocative test

Successful preclinical trials of a provocative test with αMpT^136^ have opened up prospects for conducting clinical trials. It should be emphasized that the risks to individuals in clinical trials are minimal, if any, for a number of reasons. First, it was proved in animal models that αMpT has no side effects.^136^, ^137^, ^138^ Then, αMpT, as a commercial medicine Demser/Metyrosine (Aton Pharma, Inc., USA; Demser®/Metyrosine (https://www.bauschhealth.com/Portals/25/Pdf/PI/Demser‐PI.pdf), is used for PET studying the mechanisms of dopamine neurotransmission in healthy subjects and for treating patients with overexpression of catecholamines, mainly pheochromocytoma and schizophrenia.^137^, ^139^, ^140^, ^141^

The regime of αMpT administration for dopamine depletion was developed based on the fact that at a dose of 1000 mg per day, it inhibits tyrosine hydroxylase by more than 50%.^137^, ^139^ Given that the half‐life of αMpT is 4 hours, and the maximum concentration in the blood is reached 2 hours after oral administration, adminstration of αMpT every 6 hours for 2 days should maintain its constant level in the blood. Therefore, αMpT is usually administered for 2 days in a total dose of up to 8000 mg.^139^. Based on the above data, a single administration of αMpT at a dose of 1500 mg is probably sufficient for ED of PD and should not cause side effects, despite 50% inhibition of tyrosine hydroxylase.^142^ Indeed, only at a higher dose of 4,5 g to 8,0 g, αMpT can provoke such reversible short‐term clinical manifestations as: (i) a decrease of attention, alertness, happiness; (ii) an increase of sleepiness, tension, anger, anxiety, plasma prolactin; (iii) hypokinesia, rigidity, tremor, salivation.^138^, ^142^, ^143^, ^144^

Anyway, side effects of αMPT do not persist after discontinuing medication and can be rapidly eliminated by administering L‐DOPA.^145^, ^146^ The αMpT administration even for a long time is not associated with life‐incompatible complications.

Thus, there might be a minimal risk, if any, when using αMpT as a provocative test for ED of PD.

## 6 GENERAL CONCLUSION AND PROSPECT

**FIGURE 3:**
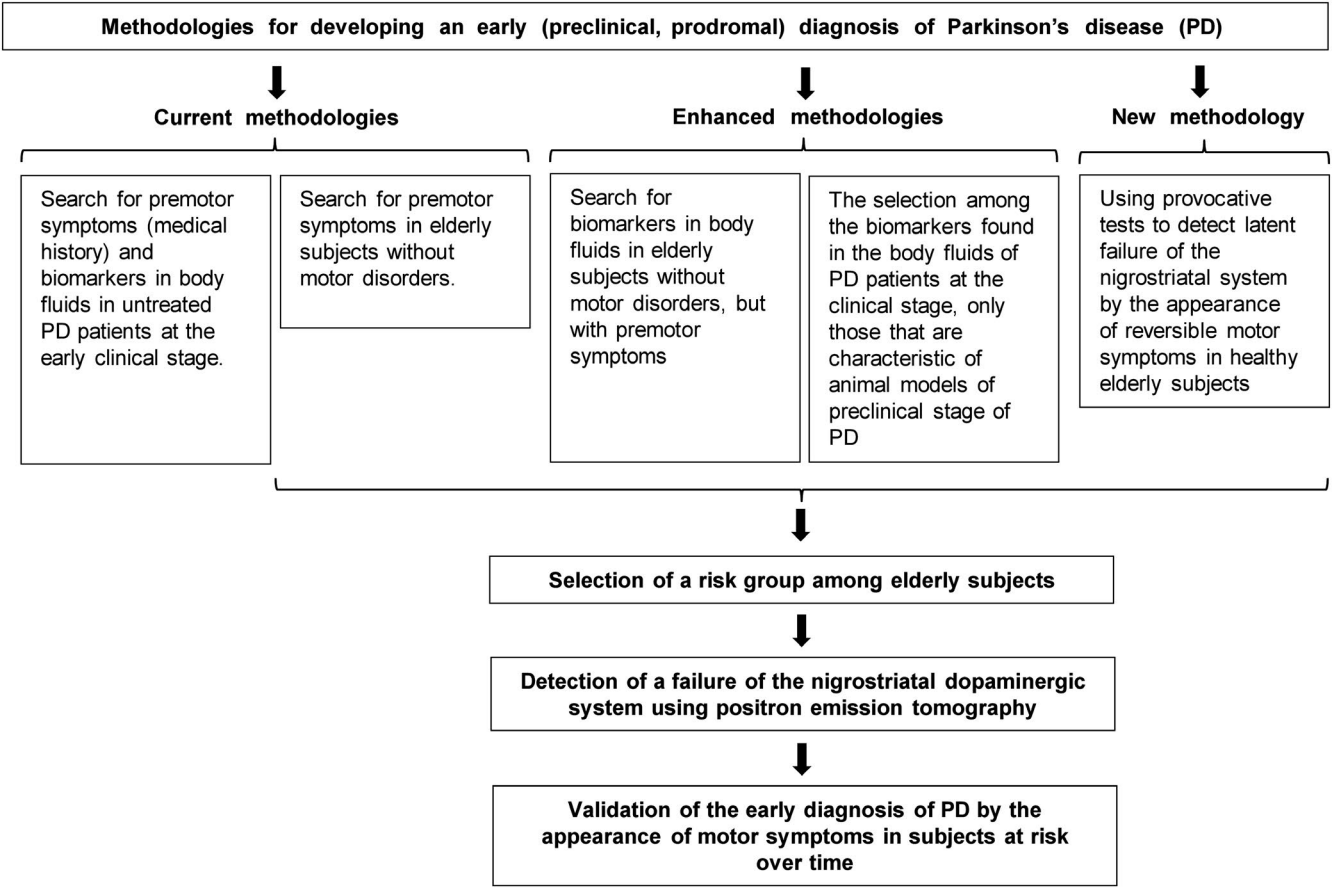
Schematic representation of methodologies for developing an early (preclinical) diagnosis of Parkinson's disease (PD)

One of the global challenges in the XXI century is the fight against socially significant neurodegenerative disease, Alzheimer disease, PD, and others. This is due to the rapidly increasing incidence of neurodegenerative disease, as well as the significant cost of treatment and rehabilitation. The low efficacy of the current symptomatic treatment of these diseases is explained by the fact that they are diagnosed by the appearance of specific dramatic symptoms many years after the onset of neurodegeneration. By this time, the majority of specific neurons have already degenerated, and the compensatory reserve of the brain is depleted. Although the development of ED of neurodegenerative disease has been considered as a priority in neurology and psychiatry in recent decades, there is still no diagnostic technology recommended for clinical use. This raises the question of the adequacy of the methodology used to solve this problem. Therefore, this review provides the critical analysis of the methodology for the development of ED of neurodegenerative disease with emphasis on PD (Figure 3).

The current methodology proceeds from the fact that PD is a systemic disease, and the degeneration of some peripheral and central neurons precedes the degeneration of nigrostriatal dopaminergic neurons—key elements of the central regulation of motor function. Therefore, this methodology is mainly based on the search for biomarkers, such as premotor symptoms and changes in BF. The former is identified by evaluating of a medical history of PD patients, or by detecting in elderly subjects with the PET‐confirmed failure of the nigrostriatal dopaminergic system. The latter are mainly searched in the CSF and blood in untreated PD patients at an early clinical stage, and they are considered as the biomarkers of the preclinical stage of PD, which is not the case (Figure 3). In fact, as follows from the PD pathogenesis, the spectrum of biomarkers should expand as the disease develops. This means that among the markers found in PD patients at the clinical stage, there may be only a small fraction of preclinical biomarkers. Moreover, most, if not all biomarkers, premotor symptoms and changes in BF found so far in PD patients are not specific to this disease.

To overcome the above problems, the following approaches are proposed to enhance the current methodology for developing ED of PD: (i) search for biomarkers in BF in elderly subjects without motor disorders, but with premotor symptoms and the PET‐confirmed failure of the nigrostriatal dopaminergic system; (ii) selection among the biomarkers found in BF in untreated PD patients at the early clinical stage biomarkers that are characteristic of animal models of the preclinical stage of PD (Figure 3). Although the use of both approaches can increase the sensitivity and specificity of the ED of PD, this diagnosis, like any other, based on the use of biomarkers, will not be fully specific. Therefore, we proposed a fundamentally new methodology for the development of ED of PD—a provocative, or challenge test (Figure 3). This test should reversibly enhance the failure of nigrostriatal dopaminergic neurons up to a threshold level at which motor disorders appear in subjects at the preclinical stage, but not in healthy individuals (Figure 2A). Such a technology was developed in animal models of PD, using αMPT, a reversible nonmetabolizable inhibitor of tyrosine hydroxylase. In contrast to the ED of PD, based on the search for premotor symptoms and changes in BF, a provocative test appears to be specific. At the current stage of developing a provocative test, it should be applied to the risk group of elderly people without motor disorders, but showing premotor symptoms and PD biomarkers in the CSF or in the blood. Then, it should be confirmed that in subjects at risk with a positive provocative test there is a failure of the nigrostriatal dopaminergic system (PET study) and, over time, motor disorders appear (Figure 3).

Rees et al.^147^ provided a critical analysis of the risks and benefits for the ED of PD. Anyway, it is tempting to assume that the development of ED of PD will encourage developing of preventive therapy. This can be a way to prolong the preclinical stage of PD and, hence, the period of normal physical, mental, and social activities.^148^

Thus, the development of ED and neuroprotective treatment of PD, even if they do not make possible to cure patients, will significantly extend the period of higher quality of life.

## CONFLICT OF INTEREST

The author declares that he has no competing interests.
